# A prospective study of tinea capitis in children: making the diagnosis easier with a dermoscope

**DOI:** 10.1186/s13256-018-1914-6

**Published:** 2018-12-28

**Authors:** Niema Aqil, Hanane BayBay, Kaoutar Moustaide, Zakia Douhi, Sara Elloudi, Fatima Zahra Mernissi

**Affiliations:** Dermatology, University Hospital Hassan II, Fez, Morocco

**Keywords:** Trichoscopy, Tinea capitis, Clinical subtype

## Abstract

**Introduction:**

Tinea capitis is a scalp infection caused by different fungi. Etiological diagnosis is based on suggestive clinical findings and confirmation depends on the fungus growth in culture. However, it is not always possible to perform this test due to lack of availability. The association of clinical and dermatoscopic findings in suspected cases of tinea capitis may help the identification of the etiological agent, facilitating precocious, specific treatment.

**Materials and method:**

We report a prospective descriptive analytical study of 34 children with tinea capitis. We performed a trichoscopic examination of all patients; only six children were able to have the mycological culture.

**Results:**

Trichoscopy was abnormal in all 34 patients; it showed hair shaft abnormalities and, in some cases, scalp disorders too. We found that the comma and corkscrew appearance was found in microsporic tinea capitis, V-shaped hair was mainly seen in inflammatory tinea capitis, scales and follicular keratosis in non-inflammatory tinea capitis, and crusts and follicular pustules in inflammatory tinea capitis. Finally, erythema was seen in trichophytic and inflammatory tinea capitis.

**Conclusion:**

We propose a classification of trichoscopic signs of tinea capitis. This classification will enable rapid diagnosis and prediction of the type of fungus before mycological culture, thus a faster and more adapted management.

Our study shows the importance of trichoscopy in the diagnosis and monitoring of tinea capitis. We suggest further prospective studies with a larger number of patients with tinea capitis, having performed mycological culture, to confirm this classification.

## Introduction

Tinea capitis (TC) is the most common dermatophytosis in children [1, 2]. In some situations, the appearance and clinical context are not obvious requiring mycological confirmation. However, the culture results can take 4 weeks to be available, which may hinder the management of these patients and increase the risk of contamination [3]. In these cases, trichoscopy can guide the diagnosis. Therefore, dermoscopic signs specific to TC must be well established.

## Materials and method

We carried out a 6-month prospective descriptive analytical study between January and June 2017, gathering the various dermoscopic signs found in children with alopecic plaques suspected of TC. We classified them according to the clinical patterns of microsporic TC, trichophytic TC, or inflammatory TC, in order to find a correlation between the dermoscopic signs and the clinical subtype. The data were saved on Excel and analyzed on the SPSS Statistics version 20 software.

## Results

**Fig. 1:**
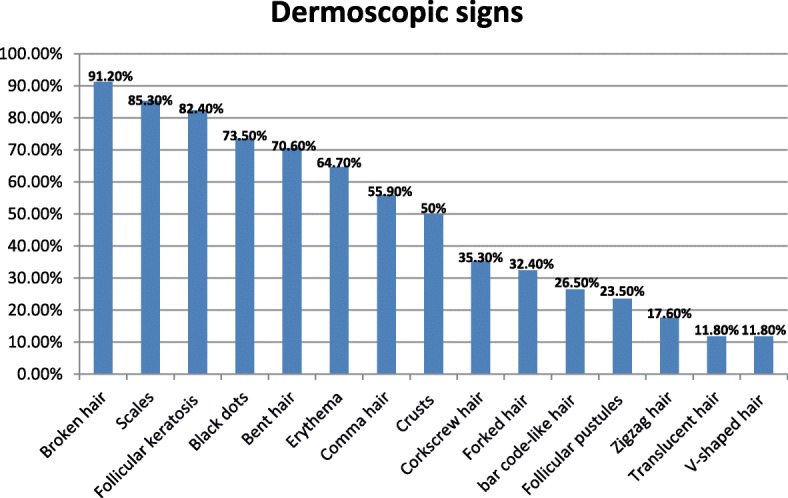
Dermoscopic signs

We collected data from a total of 34 children with alopecic plaques highly suggestive of the diagnosis of TC. The average age of our patients was 8.42 years (3–14 years). Out of the 34 children, 67.6% were boys and 32.4% girls, with a sex ratio of 2.09. Out of the 34 children, 47.51% had microsporic TC, 29.4% trichophytic TC, and 23.5% inflammatory TC (Table 1). Only six patients were able to have a mycological culture to confirm the diagnosis of TC as well as the clinical subtype; all the children received probabilistic treatment with good evolution. The other 28 patients did not have a mycological culture because of a lack of financial means. Under the dermoscope, the signs found were: broken hair (91.2%), follicular keratosis (82.4%), scales (85.3%), black dots (73.5%), bent hair (70.6%), erythema (64.7%), comma hairs (55.9%), crusts (50%), corkscrew hairs (35.3%), forked hairs (32.4%), bar code-like hair (26.5%), follicular pustules (23.5%), zigzag hair (17.6%), translucent hair (11.8%), and V-shaped hair (11.8%; Fig. 1). We did not observe a classic dermoscopic sign of alopecia areata.

**Table 1:** Patient characteristics

Total number of cases of TC	34
Average age	8.42 years (3–14)
Sex ratio	67.6% male
Microsporic TC	47.1%
Trichophytic TC	29.4%
Inflammatory TC	23.5%
Mycological sampling	18%

*TC* tinea capitis

**Fig. 2:**
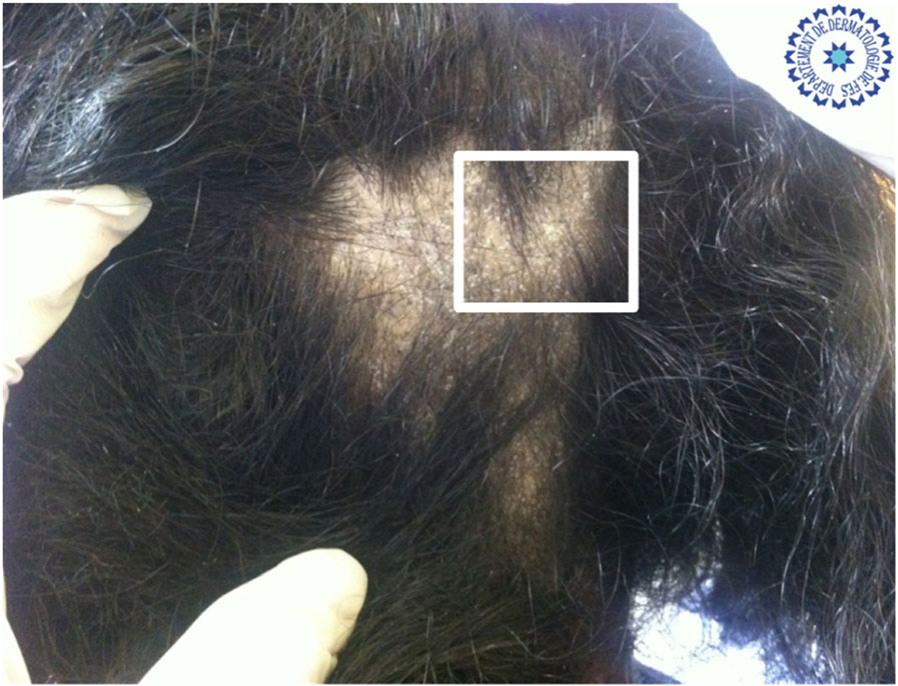
Microsporic tinea due to *Microsporum canis* with single squamous plaque of alopecia in parietal region

**Fig. 3:**
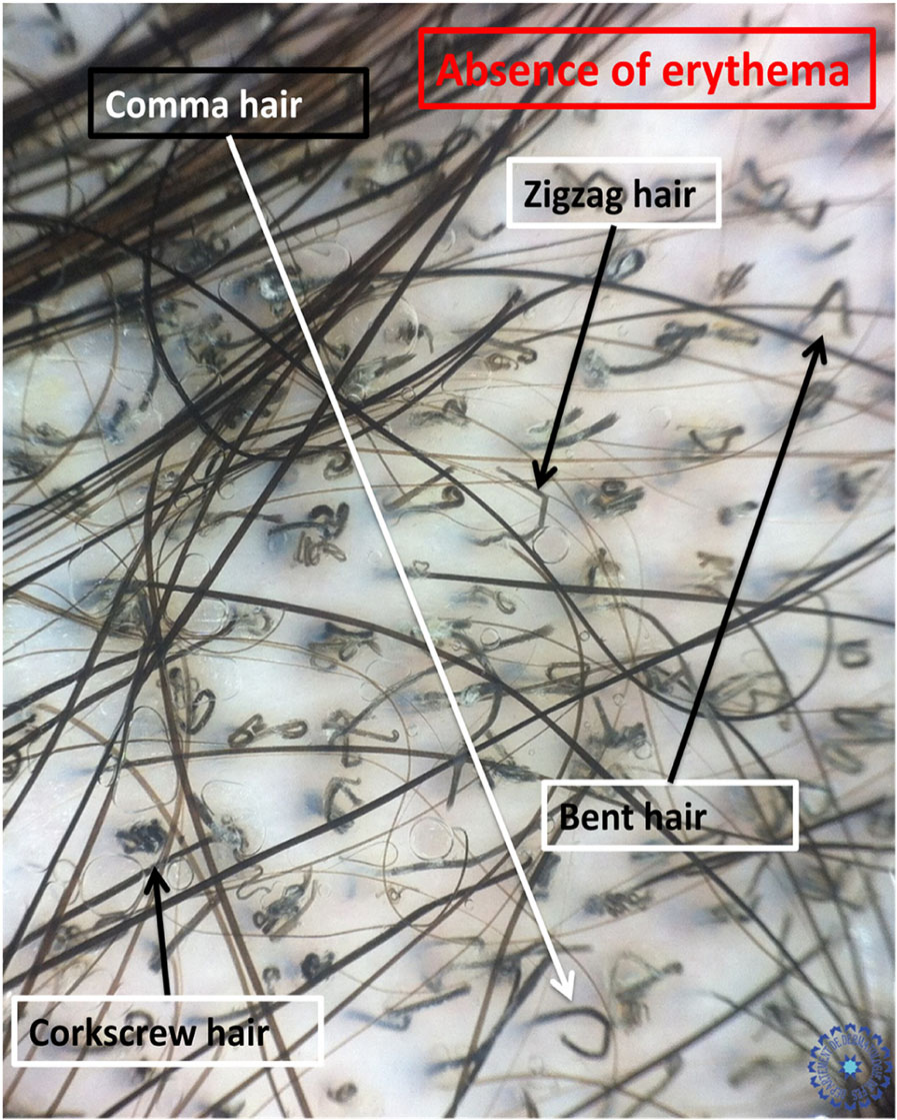
Dermoscopic image corresponding to the *inset square* of Fig. 2

**Fig. 4:**
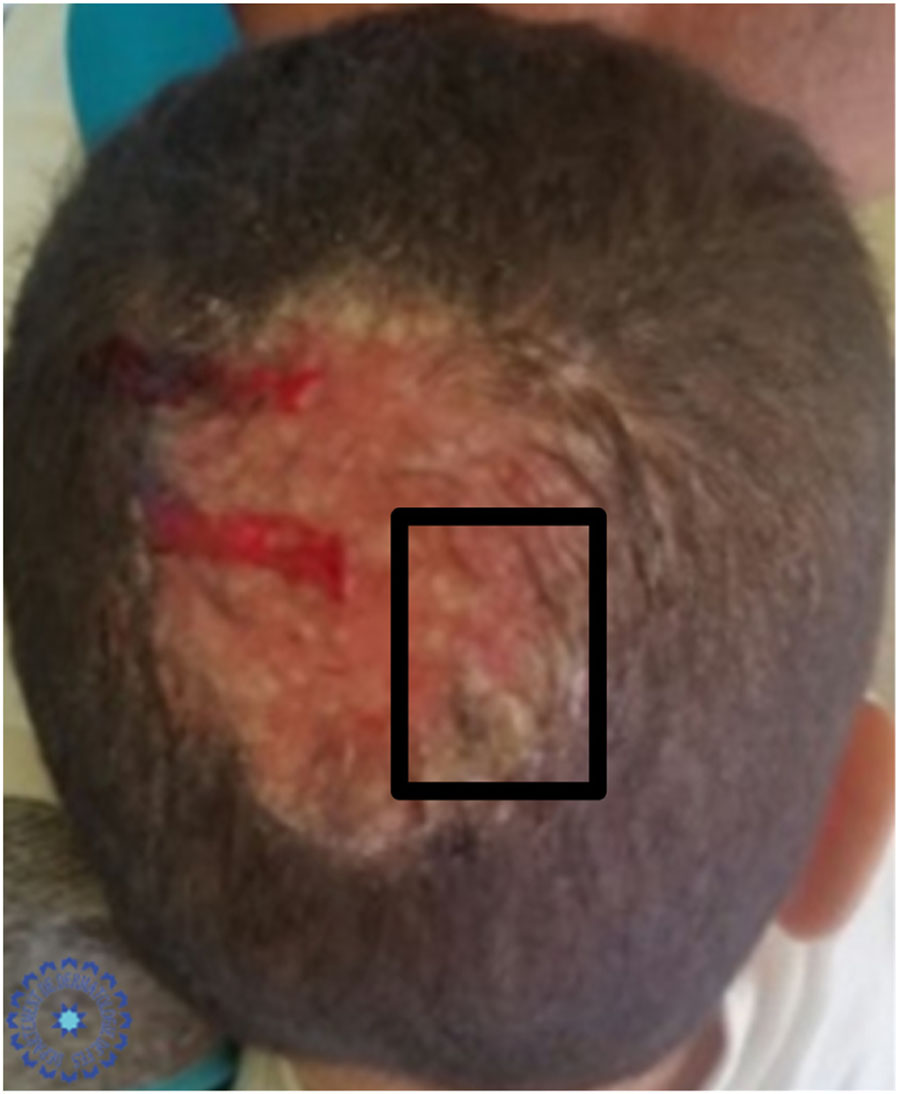
Inflammatory tinea

**Fig. 5:**
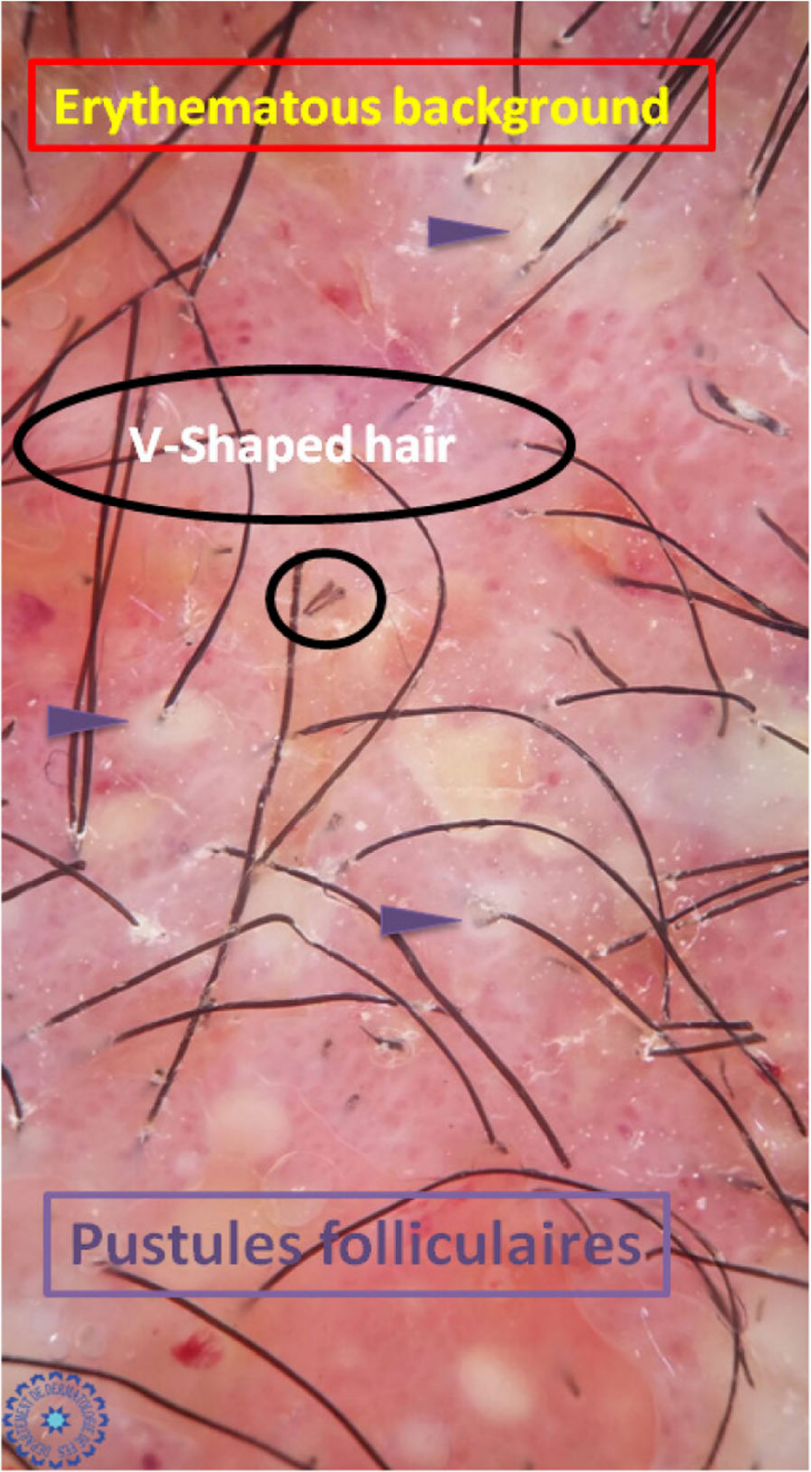
Dermoscopic image corresponding to the *inset rectangle* of Fig. 4

**Fig. 6:**
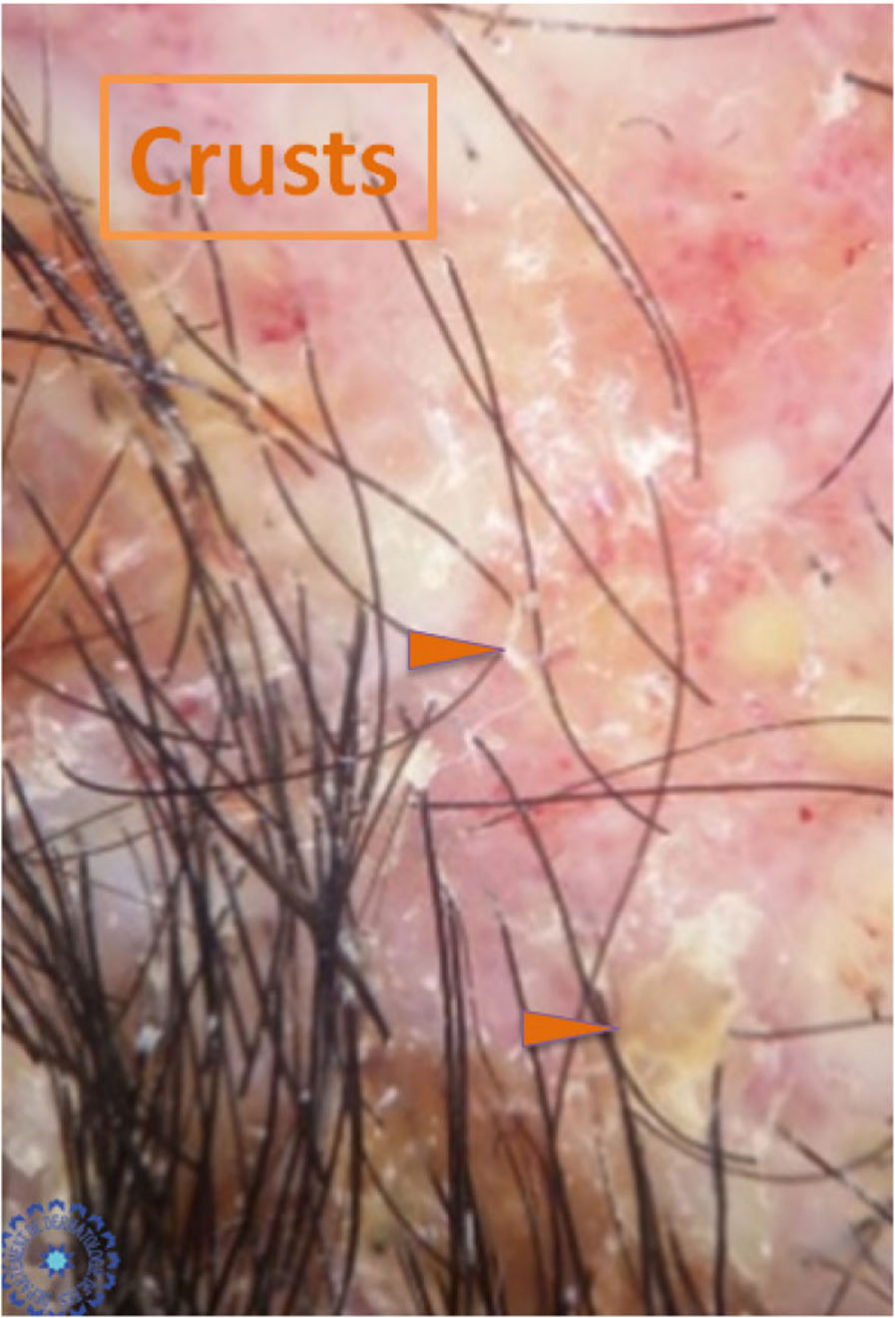
Dermoscopic image corresponding to the *inset rectangle* of Fig. 4

**Fig. 7:**
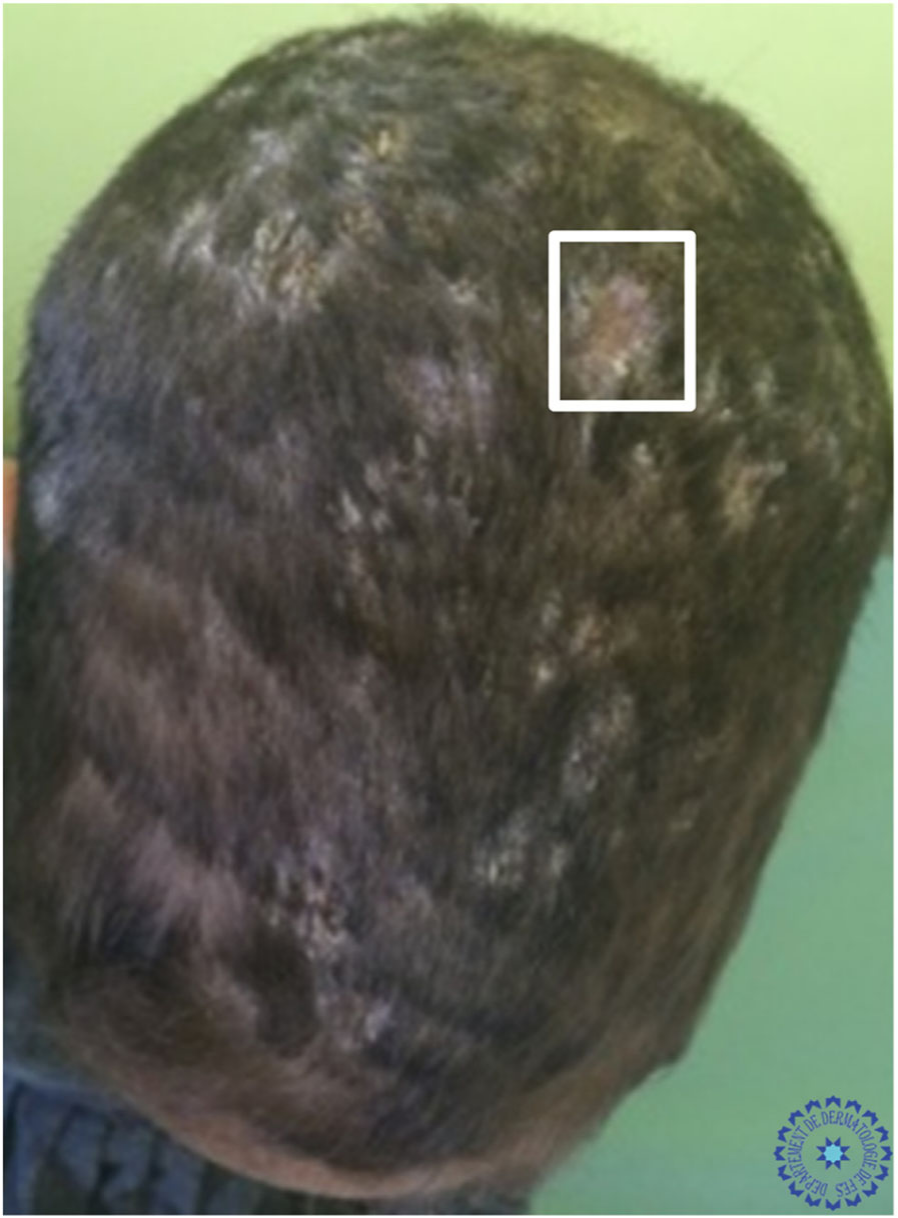
Tinea due to *Trichophyton violaceum* with diffuse small plaques of alopecia

**Fig. 8:**
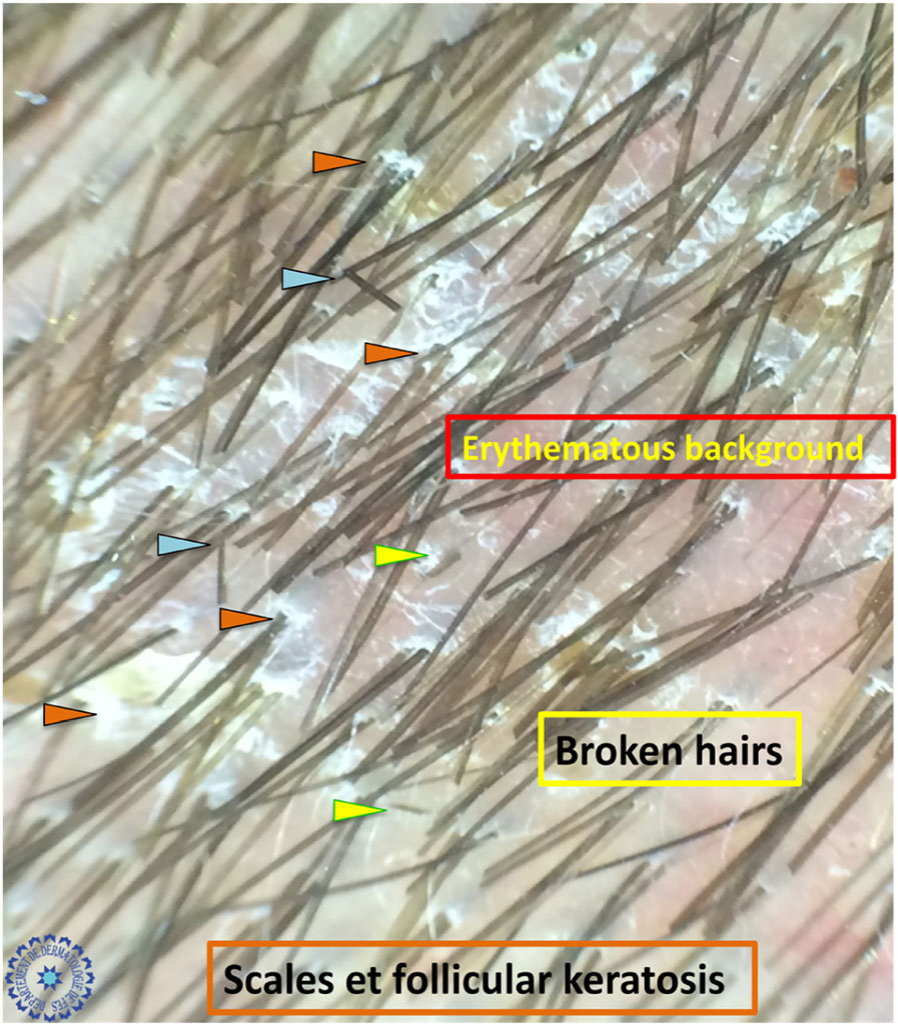
Dermoscopic image corresponding to the *inset rectangle* of Fig. 7. The orange arrowheads are pointing for scales and follicular keratosis, the yellow ones for broken hairs, the blue ones for bent hairs

Univariate analysis showed that: the sign of corkscrew hair was significantly present in female children (*p* < 0.05, *r* = 0.016), comma hair and corkscrew hair were found in microsporic TC (*p* < 0.001, *r* = 0.685 and *p* < 0.05, *r* = 0.536, respectively; Figs. 2 and 3), V-shaped hair was mainly seen in inflammatory TC (*p* < 0.05, *r* = 0.017; Figs. 4 and 5), crusts and follicular pustules in inflammatory TC (*p* < 0.05, *r* = 0.061 and *p* < 0.001, *r* = 0.000, respectively; Fig. 6), scales and follicular keratosis in non-inflammatory TC (*p* < 0.001, *r* = 0.000 and *p* < 0.05, *r* = 0.038, respectively; Figs. 7 and 8), and, finally, erythema was seen in trichophytic and inflammatory TC (*p* < 0.001, *r* = 0.889; Table 2).

**Table 2:** Univariate analysis

	Female gender (*n* = 12)	Inflammatory TC (*n* = 8)	Non-inflammatory TC (*n* = 26)	Microscopic TC (*n* = 16)	Trichophytic TC (*n* = 10)
Broken hair	92%	62.5%	100%	100%	100%
Scales*	92%	37.5%	**100%**	100%	100%
Follicular keratosis*	75%	50%	**92%**	93.7%	90%
Black dots	67%	62.5%	77%	87.5%	60%
Bent hairs	50%	75%	69%	62.5%	80%
Erythema*	50%	**100%**	54%	31%	**90%**
Comma hairs*	58%	12.5%	69%	**93.7%**	30%
Crusts*	33%	**100%**	35%	25%	50%
Corkscrew hairs*	**58%**	0%	46%	**62.5%**	20%
Forked hairs	25%	25%	35%	31%	40%
Bar code-like hair	8%	12.5%	31%	37.5%	20%
Follicular pustules*	17%	**87.5%**	4%	6%	0%
Zigzag hair	17%	0%	23%	25%	20%
Translucent hair	17%	0%	15%	12.5%	20%
V-shaped hair*	8%	**37.5%**	4%	6%	0%
***p*** Value*	**0.017**	**0.032/0.002/0.000**	**0.000/0.022**	**0.000/0.005**	**0.000**

*TC* tinea capitis

*Corkscrew hair was significantly present in girls (*p* < 0.05). Erythema, crusts, follicular pustules, and V-shaped hair were significantly present in inflammatory tinea capitis, whereas scales and follicular keratosis were mostly seen in non-inflammatory tinea capitis. Dermoscopy of microsporic tinea capitis showed significant presence of comma hair and corkscrew hair without erythema, which is in contrast to trichophytic tinea capitis where erythema was present in 90% of cases

The entries in boldface corresponds to the dermoscopic signs which *p*-value is significant

## Discussion

**Fig. 9:**
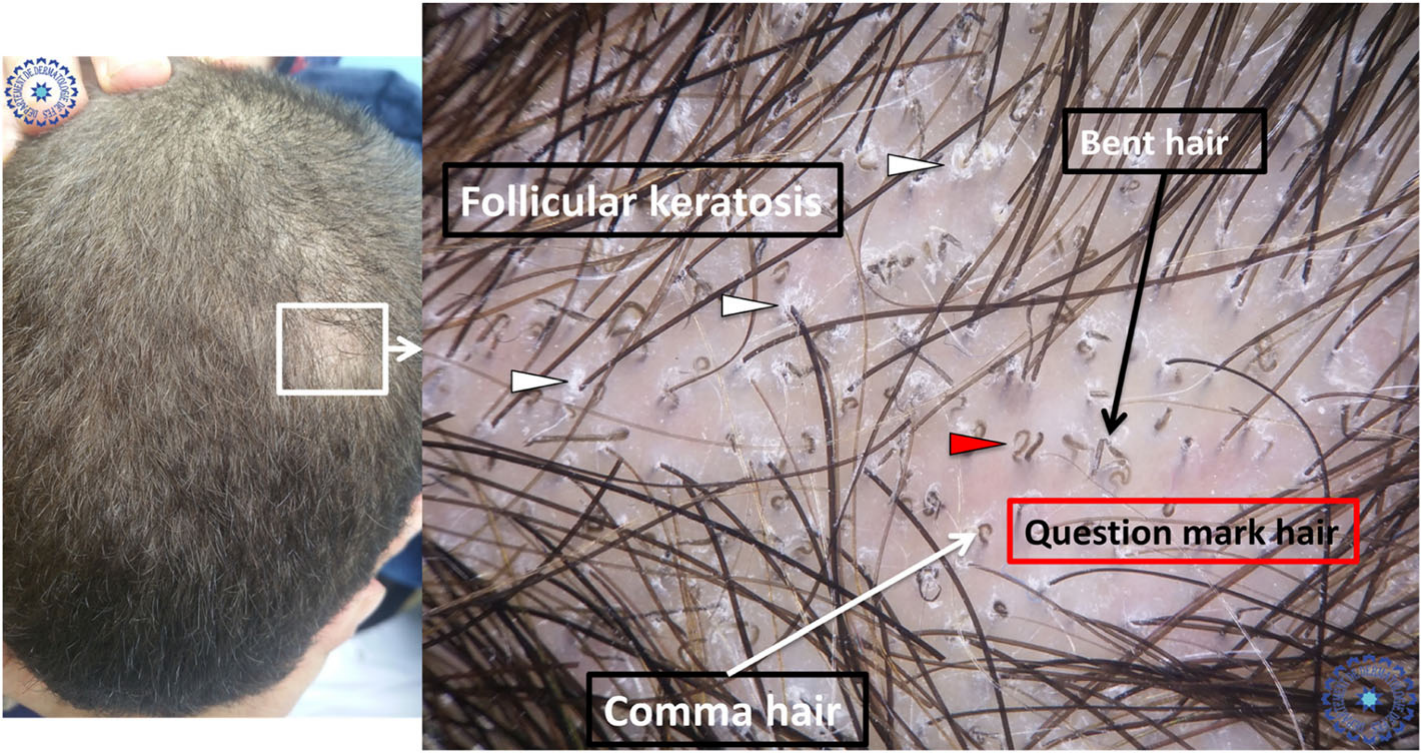
Pre-therapeutic dermoscopy showing specific signs of tinea capitis. The white arrowheads are pointing for follicular keratosis, the red ones for question mark hair

**Fig. 10:**
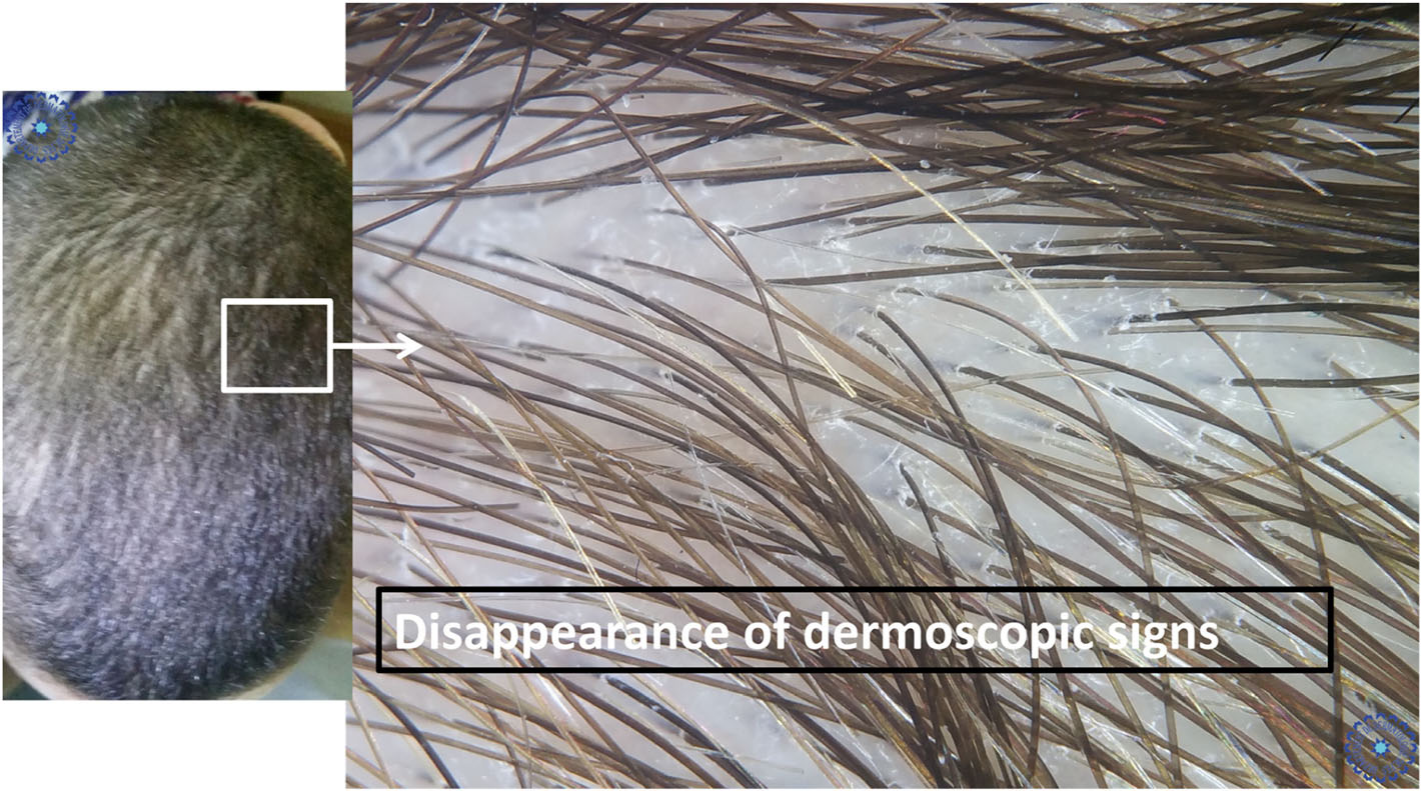
Dermoscopy 6 weeks after probabilistic treatment of the plaque shown in Fig. 11

**Fig. 11:**
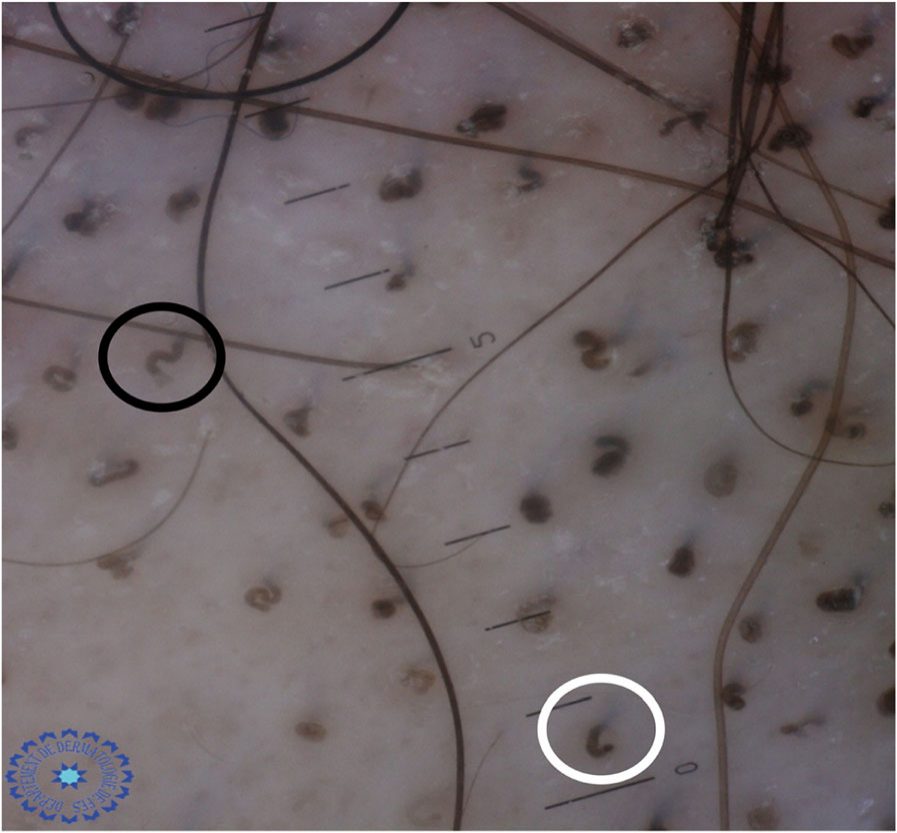
Dermoscopy showing an association of corkscrew hair (*black circle*) and comma hair (*white circle*) in the same patient due to tinea capitis

In 2008, Slowinska *et al.* described for the first time the sign of comma hair in two children with TC [4]. In 2011, Hughes *et al*. reported the sign of corkscrew hair in six black children, especially in cases of *Trichophyton soudanense* infection [5]. The authors suggested that corkscrew hair could be a variant of comma hair in black patients, or a specificity of TC due to *Trichophyton soudanense* [5]. Our study confirms the specificity of these two signs in TC since they disappeared during probabilistic treatment (Figs. 9 and 10). These two signs are often found simultaneously, with the same patient, which could be explained by the fact that our population is of an intermediate skin phototype (Fig. 11).

**Fig. 12:**
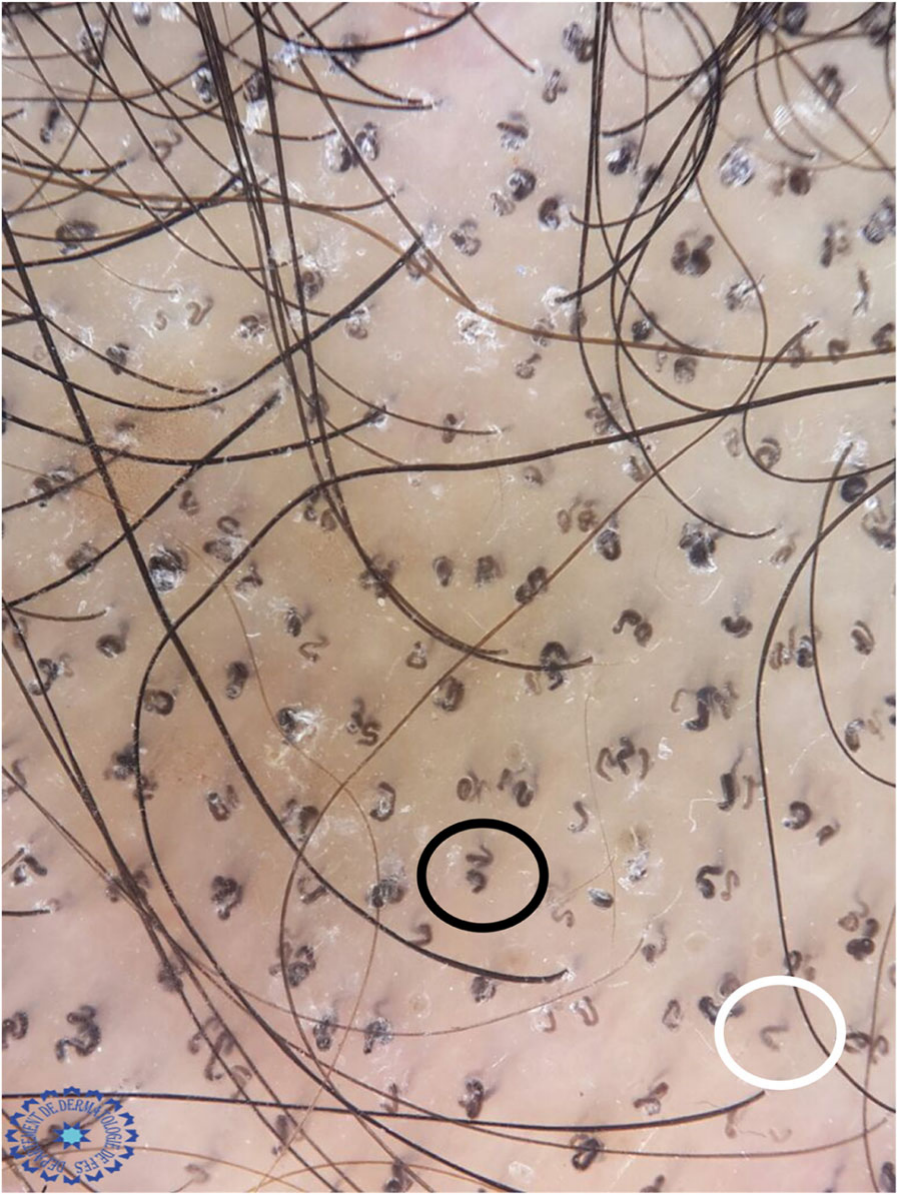
Dermoscopy showing an association of comma hair (*white circle*) and corkscrew hair (*black circle*) in a patient with tinea due to *Microsporum canis*

A study carried out in 2015 on four patients with microsporic tinea and two with trichophytic tinea showed that comma hair was specific to trichophytic tinea caused by *Trichophyton tonsurans*, while the bent hair of microsporic tinea was caused by *Microsporum canis* [6]. The Bourezane and Bourezane study of 24 patients with TC showed that infection caused by endothrix agents was responsible for abnormalities in hair shape, infection caused by ectothrix agents was responsible for abnormalities in hair color, and finally infection caused by both ectothrix and endothrix agents presented as a mixed dermoscopic pattern [7]. This is in contrast to the results of our study, where comma hairs and corkscrew hairs were significantly present in microsporic tinea (Figs. 3 and 12).

These results seem interesting when considering the choice of probabilistic treatment, especially with the emergence of species more sensitive to terbinafine than to griseofulvin. As reported in the guidelines of management of TC in England, the first-line treatment is terbinafine for trichophytic tinea, which is an allylamine that acts on the cell membrane and is fungicidal, and griseofulvin for microsporic tinea, which is a fungistatic drug that inhibits nucleic acid synthesis, arrests cell division at metaphase, and impairs synthesis of the cell wall [8, 9]. However, these studies require a broader validation; in particular, some studies have not confirmed the correlations between dermoscopic signs and the type of pathogen [10–12].

Other studies have highlighted the importance of trichoscopy in monitoring patients with TC [13, 14].

### Limitations of the study

Mycological confirmation (direct examination and culture) was not available for all patients. The authors classified patients according to the clinical pattern, in microscopic TC, trichophytic TC, or inflammatory TC, in order to make a correlation between the dermoscopic signs and the clinical subtype.

## Conclusion

In conclusion, trichoscopy is a simple, fast, and inexpensive method for diagnosing and monitoring TC in children. However, mycology remains the gold standard for diagnostic confirmation, which is also inexpensive but can take a long time. Confirmation of our results by dermoscopy/mycology correlation in large studies will allow us to treat patients only on the basis of the dermoscopic signs.
